# The Utility of Capsule Endoscopy in Patients under 50 Years of Age with Recurrent Iron Deficiency Anaemia: Is the Juice Worth the Squeeze?

**DOI:** 10.1155/2015/948574

**Published:** 2015-04-01

**Authors:** Prabhjot Singh Sidhu, Mark E. McAlindon, Kaye Drew, Reena Sidhu

**Affiliations:** Gastroenterology & Liver Unit, Royal Hallamshire Hospital, Glossop Road, Q Floor, Sheffield S10 2JF, UK

## Abstract

*Background and Aims*. The role of capsule endoscopy (CE) in the <50 years of age patients with iron deficiency anaemia (IDA) remains unclear. We aim to assess its utility in this cohort. *Methods*. All patients referred for CE for recurrent IDA were included retrospectively. Patients were divided into Group 1 (<50 years) and Group 2 (≥50 years). *Results*. There were 971 patients with recurrent IDA and 28% belonged to Group 1. The mean age was 40 years in this group with a DY of 28% (*n* = 76). Significant diagnoses included erosions and ulcers (26%; *n* = 71), small bowel (SB) angioectasia (AE) (10%; *n* = 27), SB tumours (3%; *n* = 7), Crohn's disease (3%; *n* = 7), SB bowel strictures (1%; *n* = 3), and SB varices (1%; *n* = 2). On logistic regression, the presence of diabetes (*P* = 0.02) and the use of warfarin (*P* = 0.049) was associated with increased DY. The DY in Group 2 was 38% which was significantly higher than in Group 1 (*P* = 0.02). While SB tumours were equally common in both groups, AE was commoner in Group 2 (*P* < 0.001). *Conclusion*. A significant proportion of patients <50 years are referred for CE. Although the DY is lower compared to those ≥50 years, significant pathology is found in this age group. CE is advisable in patients <50 years old with recurrent IDA and negative bidirectional endoscopies.

## 1. Introduction

Iron deficiency anaemia (IDA) still remains a common cause for referral to a gastroenterologist (up to 13%) and is often caused by chronic occult gastrointestinal bleeding [1, 2]. In patients where bidirectional endoscopy has been normal and the IDA persists, investigating the small bowel (SB) is warranted. It has been shown that, in patients with IDA, 30% of patients will have normal bidirectional endoscopies [3]. In view of this, both the American Gastroenterological Association (AGA) and the British Society of Gastroenterology (BSG) have recommended SB investigation in particular to detect lesions such as SB angioectasia (AE), Crohn's disease, and neoplasia [2, 4].

In 2001, the introduction of small bowel capsule endoscopy (SBCE) into clinical practice opened new doors in the investigation of obscure gastrointestinal bleeding (OGIB) where the cause had not yet been identified by esophagogastroduodenoscopy (OGD) and colonoscopy [5]. Today, it is the first line investigative tool for suspected SB bleeding. The DY for SBCE ranges from 34% to 92% for OGIB [6]. In anaemia exclusively, the DY is between 57% and 78% [7, 8] and, in those over 70 years of age, AE is the commonest cause of OGIB (30–40%) [9, 10]. Whilst there are many studies investigating the use of CE in OGIB, there is a paucity of studies looking specifically at the use of CE for the sole indication of IDA. The literature on the use of CE in patients < 50 years is also very limited [11].

The purpose of our study was to evaluate the utility of CE in patients under the age of 50 years presenting with recurrent IDA to a single tertiary institution in the United Kingdom. We study clinical parameters that may predict a higher DY and factors that had a subsequent impact on patient management.

## 2. Methods and Materials

### 2.1. Patients

All patients routinely referred for SBCE from June 2002 to November 2012 for investigation of recurrent IDA were included in this study. In the Sheffield Teaching Hospitals NHS Trust and its referring hospitals, haemoglobin concentrations below 13 g/dL in men and below 11 g/dL in women are references used to define anaemia. Prior to referral for CE, all patients had undergone upper and lower gastrointestinal investigation with a negative DY either locally (Sheffield Teaching Hospitals NHS Trust) or at the primary referring hospital. This study was part of the small bowel endoscopy study approved by the North Sheffield Ethics Committee (07/2308/13). Data was collected retrospectively on age, sex, indication, comorbidity, SBCE findings, DY, management change, and subsequent procedures undertaken. Significant findings on SBCE, which were deemed to be the cause of the patients clinical presentation only, were included in the diagnostic yield. Data was also collected specifically on the use of nonsteroidal anti-inflammatory (NSAIDS), warfarin, and blood transfusions.

### 2.2. Capsule Endoscopy

With patient consent, SBCE (Pillcam, Given Imaging, Yokneam Illit, Israel) was performed in patients with recurrent IDA. The procedure involved an overnight fast for 12 h after ingestion of two sachets of polyethylene glycol solution (Kleen-Prep; Norgine, Middlesex, UK). The capsule was ingested with water and 80 mg simethicone (Infacol, Forest Laboratories, Kent, UK) and data subsequently downloaded onto the computer workstation as per standard protocol [5]. All videos were analysed by either experienced consultant gastroenterologist (RS and MEM) or experienced advanced nurse practitioner (KD).

### 2.3. Statistical Analysis

The data were analysed using SPSS version 18 (SPSS Inc., Chicago, IL, USA). Regression analysis was carried out to determine which clinical factors predicted diagnosis and management change. A *P* value of less than 0.05 was considered statistically significant.

## 3. Results

### 3.1. Study Population

A total of 1324 patients were referred for SBCE for investigation of obscure gastrointestinal bleeding (OGIB). All patients had undergone both upper and lower gastrointestinal investigations (at times, multiple) without a DY. Of the 1324 patients, 73% (*n* = 971) were referred solely with recurrent IDA while the remaining were referred with overt bleeding (OB) (*n* = 353). Those with recurrent IDA were then segregated into 2 groups based on age. Group 1 was populated with patients <50 years of age while Group 2 was populated with those ≥50 years of age. The cumulative DY for patients with recurrent IDA in our study was 66%.

### 3.2. Group 1

Group 1 comprised 28% (*n* = 275) of the aforementioned 971 patients. The mean age in Group 1 was 40 years, with the age ranging from 17 to 49 years with 61% (*n* = 168) of patients being female. The DY in Group 1 was 28% (*n* = 76) with no difference between the sexes. All significant diagnoses found in this age group are tabulated in Table 1. Interestingly, 6 out of 7 SB tumours were found in females. On logistic regression, the presence of diabetes mellitus (*P* = 0.02) and the use of warfarin (*P* = 0.049) were associated with increased DY. Management was altered in 75% (*n* = 57) of patients with a DY on CE. Further procedures undertaken locally in patients with a management change included double balloon enteroscopy (DBE) (*n* = 11), push enteroscopy (PE) (*n* = 2), repeat OGD (*n* = 2), and surgery (*n* = 2). On logistic regression, the only clinical factors that influenced a management change were SBAE (*P* = 0.02).

### 3.3. Group 2

Group 2 was made up of 72% (*n* = 696) of the aforementioned 971 patients. The mean age was 68 years with the age ranging from 50 to 92 years with 58% (*n* = 407) of patients being female. The DY was 38% (*n* = 267) with no difference between the sexes. Significant diagnoses found in this age group are tabulated in Table 1. On logistic regression, the presence of haematological disease (*P* = 0.02), chronic renal disease (*P* = 0.03), and chronic liver disease (*P* = 0.04) and being transfusion dependent (*P* = 0.049) were associated with increased DY. Management was altered in 72% (*n* = 191) of patients with a DY on CE. Further procedures undertaken locally in patients with a management change included DBE (*n* = 28), PE (*n* = 20), repeat OGD (*n* = 16), colonoscopy (*n* = 8), and surgery (*n* = 7) with 17% (*n* = 32) of patients receiving APC therapy. On logistic regression, clinical factors that were associated with a management change included the presence of a comorbidity (*P* = 0.001) and SBAE (*P* < 0.001) and being transfusion dependent (*P* = 0.04).

### 3.4. Comparisons between the Two Groups

On group comparison, DY was significantly higher in Group 2 (age ≥ 50 years) (*P* = 0.002) (95% CI = 1.2 to 2.2; odds ratio = 1.6) than in Group 1. There was no significant difference in management change in patients with a DY (*P* = 0.55) or total number of procedures undertaken locally, although significantly more patients required APC therapy in Group 2 (*P* = 0.025) (95% CI = 1.3 to 75.5; odds ratio = 10.1). SB erosions and ulcers (26%; *n* = 71) were the commonest finding in Group 1 while SBAE (28%; *n* = 198) was in Group 2. In addition, Group 2 had significantly more comorbidities. There was also significantly more patients on NSAIDS (*P* = 0.041) and warfarin (*P* = 0.001) as well as being transfusion dependent (*P* = 0.01) and having had a previous transfusion (*P* = 0.001) in Group 2 as tabulated in Table 2.

## 4. Discussion

The purpose of this study was to establish the importance of CE in the investigation of younger patients with recurrent IDA of which there is limited data. Recent work by Koulaouzidis et al. has been encouraging in trying to address this shortcoming. With a DY of 25% for sinister/significant lesions in the ≤40 years of age patients, they highlight the importance of prioritising the young patient when investigating IDA with CE [11]. Their study however still had a relatively small sample size (*n* = 221). Our study builds on their findings with a much larger sample size (*n* = 971), is of an unselective nature, and is representative of CE findings in routine practice. In our study, we have demonstrated that the DY in patients <50 years was 28%. Significant pathology identified in this group included SB tumours, ulcers, and AE.

In the <50 years of age patients, SBAE is the second commonest finding after erosions and ulcers. It is found in 10% of the <50 years of age cohort and in 28% of the ≥50 year of age cohort. Although SBAE is a disease primarily inflicting the elderly, we found that SBAE also impacts as a factor in management change in both the <50 years of age patients and the ≥50 years of age patients.

SB tumours were found in 1.7% of our cohort with recurrent IDA. This is slightly lower than that of previously published work of 3.9% to 8.8% [8, 12, 13]. This result may be a reflection of CE being routinely performed in patients with recurrent IDA as compared to the stringent criteria set in other studies. In our <50 years of age patients cohort, SB tumours were found in 3% of patients which is similar to findings by Koulaouzidis et al. [11]. In addition, SB tumours were more common in females in the <50 years of age patients cohort.

In this study, we found that the DY of 66% in all patients with recurrent IDA is in keeping with DY of recent published work [8, 11, 12, 14–17]. We also found that diabetes mellitus and warfarin influenced the DY in the <50 years of age patients, whilst it was haematological disease, chronic renal failure, and chronic liver disease and being transfusion dependent that influenced DY in the ≥50 years of age patients. This is as predicted and partly explained by commonality of pathology found in these age groups. In addition, other investigators [11] including our previous work [18] have also shown that transfusions, warfarin usage, and chronic liver disease influenced DY.

This study also highlights a significant number of UGIT findings in patients undergoing CE for recurrent IDA. There has been similar reports in the literature, highlighting the importance of meticulous upper and lower gastrointestinal endoscopic examination [19, 20].

Limitations of this study included its retrospective nature, all referrals made were taken at face value, and we did not revisit the history to scrutinise any previous investigation undertaken. In addition, we did not have the menopausal status for all the females <50 years of age and our study lacked the long term follow-up data on patients which would have helped to strengthen this study. A further limitation was that there were also three reporters for all the SBCE included in this study (RS, MEM, and KD). However, we have previously demonstrated that our experienced nurse reader is equally competent as a consultant gastroenterologist in the detection of small bowel pathology and in providing a final SBCE report [21].

## 5. Conclusion

A significant proportion of patients <50 years of age with recurrent IDA were referred for CE. Although the DY is lower compared to those over 50 years, significant pathology is found in this age group. CE is advisable in patients <50 years of age with recurrent IDA and negative bidirectional endoscopies.

## Figures and Tables

**Table 1:** Distribution of all the findings on capsule endoscopy in patients <50 and ≥50 years of age with recurrent iron deficiency anaemia.

All CE findings	Group 1 (*n* = 275) (%)	Group 2 (*n* = 696) (%)	*P* value (95% CI; odds ratio)
SB ulcers and erosions	71 (26)	174 (25)	0.815 (0.8 to 1.4; 1.0)
SBAE	27 (10)	198 (28)	<0.0001 (0.2 to 0.4; 0.3)
SB tumour	7 (3)	9 (1)	0.177 (0.3 to 5.4; 2.0)
SB Crohn's disease	7 (3)	10 (1)	0.245 (0.7 to 4.7; 1.8)
Unspecified blood in the SB	6 (2)	37 (5)	0.038 (0.2 to 1.0; 0.4)
SB strictures	3 (1)	8 (1)	0.934 (0.3 to 3.6; 1.0)
SB varices	2 (1)	3 (0.4)	0.568 (0.3 to 10.2; 1.7)
Others SB findings (polyps, diverticulum, dieulafoy, endometriosis, and celiac)	6 (2)	23 (3)	0.353 (0.3 to 1.6; 0.7)
UGIT erosions and ulcers	6 (2)	46 (7)	0.008 (0.1 to 0.7; 0.3)
Unspecified blood in the UGIT	3 (1)	23 (3)	0.066 (0.1 to 1.1; 0.3)
GAVE	2 (1)	21 (3)	0.051 (0.1 to 1.0; 0.2)
Other UGIT findings (portal hypertensive gastropathy, varices, AE, tumour, and UGIT polyps)	8 (3)	31 (5)	0.273 (0.3 to 1.4; 0.6)
Colorectal lesions	4 (2)	16 (2)	0.404 (0.2 to 1.9; 0.6)

CE: capsule endoscopy; IDA: iron deficiency anaemia; 95% CI: 95% confidence interval; SB: small bowel; AE: angioectasia; UGIT: upper gastrointestinal tract; GAVE: gastric antral vascular ectasia.

**Table 2:** Distribution of anticoagulation and transfusions in the <50 and ≥50 years of age patients.

Medication	Group 1 (*n* = 275) (%)	Group 2 (*n* = 696) (%)	*P* value (95% CI; odds ratio)
Warfarin	5 (2)	74 (11)	<0.001 (0.1 to 0.4; 0.2)
Nonsteroidal anti-inflammatories	14 (5)	63 (9)	0.042 (0.3 to 1.0; 0.5)
Transfusion dependent	8 (3)	52 (8)	0.010 (0.2 to 0.8; 0.4)
Previous transfusion	6 (2)	57 (8)	0.002 (0.1 to 0.6; 0.3)

## Abbreviations

CE: Capsule endoscopy
SBCE: Small bowel capsule endoscopy
OGIB: Obscure gastrointestinal bleeding
DBE: Double balloon enteroscopy
APC: Argon plasma coagulation
GI: Gastrointestinal
OR: Odds ratio
CI: Confidence interval.
